# Alleviate oxidative stress in diabetic retinopathy: antioxidant therapeutic strategies

**DOI:** 10.1080/13510002.2023.2272386

**Published:** 2023-12-02

**Authors:** Jie Gao, Liming Tao, Zhengxuan Jiang

**Affiliations:** Department of Ophthalmology, The Second Affiliated Hospital of Anhui Medical University, Hefei, People’s Republic of China

**Keywords:** Diabetic retinopathy, Oxidative stress, ROS, Antioxidant, therapeutic strategies, hyperglycemia, microvascular complication, imbalance

## Abstract

**Objectives:**

This review outlines the function of oxidative stress in DR and discusses therapeutic strategies to treat DR with antioxidants.

**Methods:**

Published papers on oxidative stress in DR and therapeutic strategies to treat DR with antioxidants were collected and reviewed via database searching on PubMed.

**Results:**

The abnormal development of DR is a complicated process. The pathogenesis of DR has been reported to involve oxidative stress, despite the fact that the mechanisms underlying this are still not fully understood. Excessive reactive oxygen species (ROS) accumulation can damage retina, eventually leading to DR. Increasing evidence have demonstrated that antioxidant therapy can alleviate the degeneration of retinal capillaries in DR.

**Conclusion:**

Oxidative stress can play an important contributor in the pathogenesis of DR. Furthermore, animal experiments have shown that antioxidants are a beneficial therapy for treating DR, but more clinical trial data is needed.

## 1. Introduction

Diabetes is now considered a rapidly expanding worldwide health emergency in the twenty-first century, with a global adult prevalence of 8.8% of the world's population in 2017 and a further increase to 9.9% projected by 2045 [1]. According to the report of World Health Organization (WHO), DR is responsible for at least 5% of the 37 million instances of blindness globally, making it the primary cause of visual impairment in adults. Furthermore, the WHO predicts that diabetes will be the 7th leading cause of mortality by 2030 [2, 3]. Diabetes can be classified into two types: type 1 mellitus (T1DM) and type 2 diabetes mellitus (T2DM) [4]. DR is a severe microvascular complication of both T1DM and T2DM, which has been linked to the production of free radicals, oxidative stress and chronic inflammation [5].

Elevated oxidative stress is becoming more widely recognized of a critical factor in the pathogenesis of diabetes and its complications [6]. Reactive oxygen species (ROS) are produced in excess, which results in oxidative stress, a biological process that aids in the etiology of many diseases and associated complications. ROS cause cell injury by interacting with various cellular components. Fortunately, organisms are shielded against ROS by an antioxidant mechanism [7–9]. Despite the retina's efficient antioxidant defense mechanism, which consists of free radical scavengers and antioxidant enzymes, sustained hyperglycemia leads to a decrease in these defenses and an increase in oxidative stress [10–12]. Multiple antioxidants are currently being tested in clinical trials, but the outcomes remain inconclusive. Although this topic has been discussed in previous reviews, this review highlights the most recent antioxidant treatments based on what our research team has published on DR in recent years and updates readers on how oxidative stress contributes to the development of DR.

## 2. Diabetic retinopathy

**Figure 1.**
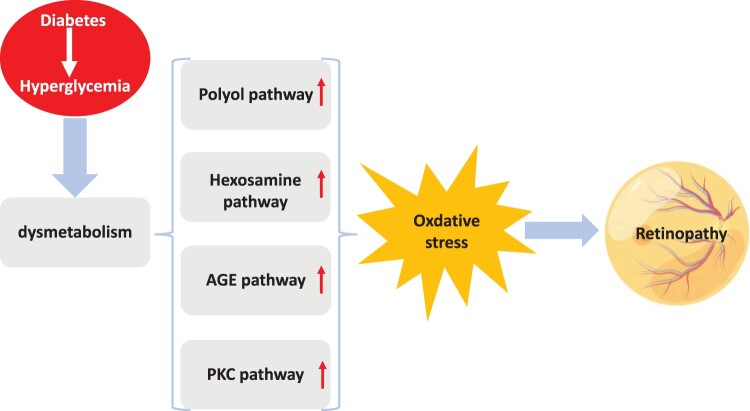
Diabetes metabolic dysfunction caused by hyperglycemia. The primary pathways contributing to the pathogenesis of retinopathy by promoting oxidative stress and generating ROS include the polyol pathway, hexosamine biosynthesis pathway, formation of advanced glycation end products (AGEs), and activation of protein kinase C (PKC).

The primary cause of vision loss and blindness in individuals of working age is diabetic retinopathy (DR), as well as one of the most serious microvascular consequences of diabetes mellitus (DM) [13]. According to clinical and research findings, hyperglycemia represents the primary cause of complications in diabetes [14]. By 2045, the number of persons worldwide with DR is projected to grow to 160.50 million from 129.84 million in 2030 [15]. The DR is a serious global public health and economic problem as a result of this disturbing future [16]. DR is clinically characterized by damage to the retinal microvasculature, which can result in hemorrhaging, angiogenesis, microaneurysms, and even retinal detachment and blindness in the worst cases [17]. Despite numerous studies, the precise mechanisms by which hyperglycemia contributes to retinal pathology still obscure. It has been postulated that aberrations in various metabolic pathways are significantly linked to the development of hyperglycemia-mediated retinopathy. As depicted in Figure 1, four main pathways – the polyol pathway, the advanced glycation end-products (AGE) pathway, the hexosamine biosynthetic pathway (HBP), and the protein kinase C (PKC) pathway – are thought to be responsible for the retinal damage brought on by hyperglycemia [18, 19]. However, clinical trials testing inhibitors of each of these distinct pathways have produced disappointing outcomes in terms of slowing the effects of DR [17].

The treatment for DR is dependent on its stage or type [20]. DR is a progressive disease that progresses in stages and can be defined as non-proliferative (NPDR) and proliferative (PDR) types with or without macular oedema depending on whether retinal neovascularization is present [21, 22]. Diabetic macular edema (DME) is another complication of DR, which is induced by a breakdown of the external blood-retinal barrier (BRB) and subsequent fluid accumulation [23]. Recent studies have identified three highly effective treatments for DR that can significantly reduce vision loss caused by the disease. If treated before the retina is seriously injured, even those with advanced DR have a 90% chance of preserving their eyesight [7]. The normative treatment for DR commonly involves anti-vascular endothelial growth factor (VEGF) drugs or corticosteroids. Anti-vascular endothelial growth factor (VEGF) drugs or corticosteroids are commonly used as the standard treatment for DR. In the clinic, intravitreal injections of anti-VEGF medications such bevacizumab, aflibercept, and ranibizumab are administered [24]. Combination therapy including laser photocoagulation and vitreoretinal surgery may be used for different stages in DR [25]. However, while these treatments may alleviate symptoms, it is crucial to identify novel therapeutic targets for the prevention and treatment of DR progression.

## 3. Oxidative stress and ROS

Over the past 40 years, various of medical fields, including ophthalmology, have extensively studied the concept of ‘oxidative stress’ [26]. Oxidative stress specifically refers to an imbalance between free radicals, which are highly reactive molecules produced in our bodies through a variety of pathways, and intrinsic antioxidant capacity [27]. Oxidative stress is extremely complicated, connected to other types of stress, and has impacts on different cell types [28]. Therefore, antioxidant therapy may be beneficial in preventing retinal damage, as oxidative stress plays a significant role in the pathogenesis of DR [29].

**Figure 2.**
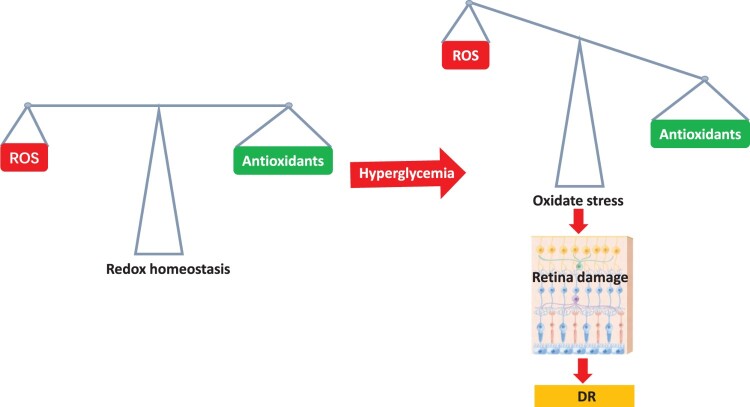
Diabetes disrupts Redox balance in retina induced by hyperglycemia. There exists a delicate balance between the formation of free radicals and the antioxidant defense mechanisms under normal physiological conditions. Hyperglycemia induced by diabetes disrupts this equilibrium by promoting ROS production while simultaneously suppressing retinal antioxidant response.

Currently, ROS involvement is well acknowledged as a crucial causal element in the rise of DR. [30]. Chronic hyperglycemia makes microvessels in the retina more susceptible to oxidative stress, which increases the production of ROS [5, 31]. ROS are free radicals, which are extremely unstable and reactive oxidant molecules due to the presence of an extra electron. In order to restore stability, they obtain electrons from nearby molecules, resulting in the formation of an oxidative chain. [32]. Notably, free radicals are constantly produced during normal metabolic processes, and intracellular antioxidants work to balance the production and neutralization of these ROS. However, pathological conditions can disrupt this delicate equilibrium by either increasing ROS production or decreasing their removal, resulting in an excessive bioavailability of ROS (Figure 2).

As is apparent from the above results, DR is associated with elevated ROS levels. Reduced antioxidant enzyme activity, hyperglycemia-induced metabolic pathways, mitochondrial oxidative phosphorylation, and nicotinamide adenine dinucleotide phosphate- (NADPH-) oxidase (Nox) are all common contributors to ROS elevation. [33, 34]. In hyperglycemic conditions, the excessive production of superoxide (including superoxide radical anion, hydroxyl radical, H_2_O_2_ and singlet oxygen) can activate various abnormal biochemical metabolic pathways due to mitochondria being the main endogenous source of ROS [35, 36]. Extensive scientific reports have also shown, due to alterations in the biomechanisms that are involved in the recovery and progression of microvascular complications, ROS play a crucial role in the pathological processes that occur in the retina of DR [32, 37]. ROS are capable of causing the development of DR, but are not the only cause, e.g. ROS interact with other pathways and crosstalk with each other. Therefore, more critical points need to be taken into account to prevent or treat these diseases by combining ROS antioxidants with other drugs. Herein, the alleviation elimination of ROS formation presents a potential antioxidative approach for DR-induced blindness.

## 4. Antioxidant therapeutic strategies

In prior chapters, we have shown that oxidative stress is crucial for the development of DR, and excessive accumulation of ROS can damage the tissue surrounding and within retinal vessels, ultimately resulting in DR. Therefore, by preventing the generation of ROS, eliminating free radicals, or strengthening the antioxidant defense system, antioxidants are postulated to mitigate aberrant metabolism and decelerate the progression of DR [19]. Thus, these elements are the focus for treating DR. The classification of various antioxidant compounds in the prevention and treatment of oxidative stress in DR is covered in the section that follows.

### 4.1. Drugs

A specific inhibitor of branched chain amino transferase (BCATc), gabapentin is a leucine analogue. Gabapentin, via suppressing glutamate excitotoxicity, has the potential to be a therapeutic treatment for reducing oxidative stress and apoptosis in diabetic retinas, which may be achieved through its ability to decrease branched chain amino acids (BCAAs) levels via inhibition of BCATc, leading to reduced glutamate synthesis and increased in the rate of glutamate oxidation [38]. Ola et al. suggested that gabapentin-stimulated glutamate clearance improved retinal cell apoptosis and oxidative stress in diabetic rats [39].

A peroxisome proliferator-activated receptor type α (PPAR-α) agonist called fenofibrate is employed for the treatment of hypertriglyceridemia and hyperlipidemia in the clinic. Fenofibrate has been demonstrated to considerably halt the progression of DR and decrease the need for laser therapy in DR [40]. Hsu et al. have confirmed that fenofibrate can decelerate the advancement in DR via regulating signaling pathways involving apoptosis and stress [41].

A mixture called sulodexide, which combines dermatan sulfate with low-molecular-weight heparin, exhibits potential as a target of therapy for a number of vascular disorders [42]. In vivo, sulodexide has been shown to enhance the retinal arterioles’ glycocalyx, decrease vascular permeability and inhibit retinal neovascularization, indicating its potential to act as a promising DR therapeutic treatment [43, 44]. Strikingly, sulodexide protected from hyperglycemia-induced ROS vascular wall production and endothelial dysfunction in a dependent on concentration manner. From a clinical perspective, sulodexide could be used to decrease oxidative stress in treating DR [45].

Bilobalide, the pharmacologically active sesquiterpene trilactone found in *Ginkgo biloba* leaves, has been shown to exhibit various pharmacological effects such as antioxidant, anti-inflammatory and anti-cancer properties [46]. In diabetic rats induced by STZ, several researches indicated that bilobalide protects retinal ganglion cells (RGCs) and exerts an antihyperglycemic effect [47, 48]. Sun et al. then suggested that bilobalide increased erythroid 2-related factor 2 (Nrf2) and Heme oxygenase-1(HO-1) expression while inhibiting oxidative stress by reducing inflammatory mediators. Notably, all products of HO-1 activity are biologically active, and the products include biliverdin and bilirubin, both of which are putative antioxidants, as well as CO, which is also biologically active. Therefore, bilobalide may effectively against DR by mitigating oxidative stress and inflammation [49].

Carvedilol (CAR), an adrenergic receptor blocking agent, is widely used in the management of cardiovascular diseases. CAR has been shown to possess anti-oxidative stress and anti-inflammatory properties [50]. For instance, CAR inhibits inflammatory and oxidative mediators to prevent pancreatic cell damage and the development of T1DM in mice. An example is that CAR prevents pancreatic β cell damage and T1DM development in mice by inhibiting inflammatory and oxidative mediators [51]. Zhang and co. reported that CAR reduces oxidative stress and apoptosis induced by HG in retinal pigment epithelial (RPE) cells through activation of the Nrf2/ARE pathway. Therefore, it is a prospective molecule for DR treatment in the future [52].

Conbercept is a novel anti-vascular endothelial growth factor (VEGF) drug that has been independently developed by China in recent years [53]. Conbercept's effectiveness as an adjuvant to vitrectomy is mostly attributable to its capacity to target various members of VEGF family (VEGF-A, B, C, and PIGF), preventing the development of neovascularization and decreasing vascular permeability in the retina [54]. Xia et al. demonstrated that conbercept exerts prominent suppressive effects on oxidative stress in mice with DR, providing further insight into the molecular mechanisms underlying its therapeutic efficacy [55].

Scutellarin (SC), a natural flavonoid, serves as the chemical biomarker for quality control of certain traditional Chinese medicines such as Scutellariae barbatae Herba and Erigerontis Berba listed in China Pharmacopoeia. The Scutellariae barbatae Herba has been reported to possess antioxidant property [56]. Additionally, an earlier study revealed that an ethanol extract from this herb attenuated DR development [57]. Mei et al. demonstrated that SC mitigates BRB breakdown by inhibiting retinal inflammatory responses and subsequent oxidative stress damage triggered by hyperglycemia-activated microglia cells during DR progression [58].

Cilostazol, a phosphodiesterase 3 (PDE3) inhibitor, is indicated for the treatment of peripheral vascular occlusive disorders as well as intermittent claudication in diabetes patients [59]. Previous research has indicated that cilostazol may exert effective anti-inflammatory and anti-oxidative functions through multiple pathways. Yeh et al. indicated that cilostazol can mitigate oxidative stress and inflammatory respones during the development of DR. Thus, cilostazol may be valuable for preventing the growth of DR [60].

### 4.2. Phytoestrogens

In RPE cells, the bioactive flavonoid component quercetin has been demonstrated to exert anti-inflammatory and protective effects against oxidative stress-induced cell apoptosis [61]. This compound may efficiently prevent diabetic retinal neurodegeneration and oxidative stress damage by increasing the levels of glutathione (GSH), superoxide dismutase (SOD), and catalase in rat retinas with diabetes and suppressing the expression of NF-B and Caspase-3 [62]. Our research team discovered ultrasmall Fe-Quercetin nanozymes particles by combining quercetin with low-toxic iron ions to prevent and delay the development and progression of DR. It possesses strong ROS scavenging capabilities, effective relief of early DR symptoms, suppression of DR progression and multitarget-specific beneficial effects for DR suggest their potential for clinical translation [63].

A coumestan isoflavone known as coumestrol (CMS) is vital for treating diseases linked to estrogen, like DM [64]. The effectiveness of 10-hydroxy-CMS as an antihyperlipidemic drug in STZ-induced diabetes has been demonstrated in a prior investigation [65]. Subsequently, CMS alleviated DR by reducing inflammation, apoptosis, and oxidative stress via SIRT1 activation, according to Xu et al [66].

### 4.3. Polyphenols

Dihydromyricetin (DMY), a flavonoid found in Ampelopsis grossedentata, has antioxidant, anti-angiogenic, and anti-inflammatory properties that suggest it may be beneficial for inhibiting the progression of diabetes and treating related complications [67]. A previous study has shown that DHM protects against HG-induced oxidative stress and apoptotic damage in RPE cell line cells via inhibiting miR-34a expression [68]. Our laboratory has reported for the first-ever the construction of ultra-small Fe-DMY nanoparticles (Fe-DMY NCPs), which are composed of DMY combined with low-toxicity iron ions. Fe-DMY NCPs protect vascular endothelial cells from oxidative stress induced by high glucose levels, scavenge excess ROS, and ameliorate the pathogenic symptoms of DR. The Poldip2-Nox4-H_2_O_2_ signaling pathway can be inhibited by Fe-DMY NCPs, which can also downregulate important vascular function markers. These results imply that Fe-DMY NCPs possess the potential to be a new multimeric drug for DR therapy, acting as a safe and efficient antioxidant and micro angio-protective agent [69].

Tricin, a flavone derived from rice bran, has demonstrated diverse bioeffects encompassing anti-cancer, anti-atherogenic and anti-inflammatory properties [70]. Tricin reduces ROS production in endothelial cells, leading to downregulation of vascular endothelial growth factor receptor 2 (VEGFR2) signaling and inhibition of HIF-1α accumulation in tumor cells, resulting in decreased expression of VEGF [71]. Yang et al. discovered that tricin inhibit angiogenesis and oxidative stress in retinal epithelial cells of DR rats by reinforces the Sestrin2/Nrf2 signaling pathway [72].

Curcumin, an active phytochemical found in *Curcuma Longa*, exhibits outstanding antioxidant effect by suppressing HIF-1 and inhibiting hypoxia-induced angiogenesis [73]. Numerous animal studies have demonstrated that curcumin can activate the Nrf2 antioxidant pathway, which mitigates cellular oxidative damage [74]. Moreover, curcumin may reduce diabetic retinal damage by acting as an antioxidant, as reported by Xie et al. Through its antioxidant, hypoglycemic and anti-inflammatory properties, it may have therapeutic promise for preventing the development of DR [75].

Proanthocyanidins (PACs) are polyphenolic compounds present in a variety of plant and fruit extracts that have been shown to exhibit effective antioxidant, anti-inflammatory and immunosuppressive properties [76]. By increasing the ratio of B-cell lymphoma-2 (Bcl-2)/Bcl-2-associated X (Bax), lowering caspase cleavage, attenuating ROS production and decreasing the rate of apoptosis, the research has shown that PACs might defend the RPE from vitamin A dimer-mediated photooxidation damage [77]. In diabetic rats, PAC treatment decreased apoptosis and enhanced retinal structure [78]. Moreover, Li et al. demonstrated that PACs have the potential to protect RPE cells from high glucose-induced damage by inhibiting ROS generation, indicating their candidacy for DR management [79].

Licorice root contains a bioactive flavonoid named isoliquiritigenin (ISL), which has been shown to possess anti-inflammatory and antioxidant properties. Moreover, Alzahrani et al. indicated that ISL would be a useful therapeutic approach for both DR prevention and treatment. Additionally, it appears that the miR-195/SIRT-1/NF-B pathways are involved in mediating the positive effects of ISL therapy [80].

Rhaponticin (RN), a natural polyphenolic flavonoid, is recognized for its pharmacological properties, particularly as an anti-diabetic agent. Moreover, the RN metabolite rhapontigenin exhibits antioxidant activity and protects against oxidative stress-induced cell injury. Shi et al revealed that treatment of RN to diabetic rats prevented the development of diabetic retinal alterations via exerting antioxidant, anti-inflammatory and hypoglycemic properties [81].

Kaempferol belongs to the flavonol subclass, which is abudant in numerous traditional herbs and edible plants. Kaempferol has been identified as a potent scavenger of superoxide, and its capacity to lower superoxide levels concentrations may have a significant impact on its antioxidant properties. This is due to the fact that the formation of superoxide radical anion is a necessary step in the generation of the majority of reactive oxygen and nitrogen species that contribute to oxidative stress [82]. Du et al. revealed that kaempferol possesses antioxidant and antiapoptotic properties, which can protect from damage induced by oxidative stress in human RPE cells. These results suggest that kaempferol may be used therapeutically to prevent and treat DR mediated by oxidative stress [83].

### 4.4. Lipids

Oxidized low-density lipoprotein is primarily composed of lysophosphatidylcholine (LPC) [84]. LPC induces endothelial cell dysfunction by generating ROS in the vascular endothelium and triggering oxidative stress through elevating free Ca^2+^ concentration in the cytoplasm of muscle cells, macrophages, and leukocytes. [85]. Plasma levels of LPC, which prevents glucose absorption in heart, muscle and liver tissues and can result in postprandial hyperglycemia, were significantly elevated in DR [86]. Furthermore, the research has demonstrated that LPC counteracts the beneficial impacts of bone marrow mesenchymal stem cells (BMSCs) on oxidative stress damage in human retinal microvascular endothelial cells (HRMECs) through TLR4/NF-κB signaling pathway [87].

Long-chain n-3 fatty acids, also referred to as omega-3 fatty acids, are essential for the healthy development of neurological structures in both the brain and eye. Previous studies have demonstrated the potential benefits of supplementation with n-3 fatty acid in inhibiting ocular diseases, probably through their anti-inflammatory and antioxidant properties. According to Suzumura et al., n-3 fatty acids may be useful for both preclinical DR prevention and DR progression slowing [88].

### 4.5. Vitamins

Vitamin B9, often known as Folic acid (FA), acts as a crucial player in single-carbon metabolism that is indispensable for multiple biological processes. A study has shown that serum homocysteine (hcy) levels in patients with T2D are negatively correlated with FA, indicating that FA may have beneficial effects in both the prevention and treatment of DR through alleviating hyperhomocysteinemia (Hhcy) [89]. Lei et al. further suggested that FA has the potential to serve as a therapeutic drug for DR by inhibiting oxidative stress, inflammation and angiogenesis [90].

Vitamin C can shield against the onset of DR by avoiding lipid peroxidation, scavenging free radicals and lowering the formation of ROS. As a result, taking vitamin C supplements is thought to be effective for the relief of DR. Research has shown that vitamin C improves retinal vascular endothelial dysfunction and reduces leukocyte adhesion in diabetic rats [19].

Vitamin D, widely recognized as an important component for bone metabolism, has also been in studies to possess potent antioxidant properties [91]. Previous studies have shown that vitamin D treatment with vitamin D can decrease oxidative stress damage in diabetic rats and DR under high glucose conditions, as well as confer cellular and tissue protection against it. Furthermore, a study has indicated that vitamin D possesses anti-inflammatory properties and enhances antioxidant defense, which may protect the retina [92].

Vitamin E, the main antioxidant in lipids, has been demonstrated to scavenge free radicals outside cells through non-enzymatic mechanisms in DR. Further research has demonstrated that vitamin E may serve as an adjunctive therapy for individuals with DR by reducing oxidative stress and possibly preventing complications of diabetes mellitus in the future [93].

### 4.6. Carotenoids

Zeaxanthin, a dietary carotenoid found only in the retina, has been demonstrated to reduce inflammation and oxidative stress in the retina of diabetic rats. Furthermore, zeaxanthin can prevent neovascularization caused by VEGF in the human retina through activating a Nox4-dependent mechanism [18].

Astaxanthin is a xanthophyll carotenoid that exhibits potent in vitro and in vivo antioxidant capacity. Notably, retinal cells are shielded from damage caused by oxidative stress through the protective effects of astaxanthin [94]. By activating antioxidant enzymes via the PI3 K/Akt/Nrf2 pathway, astaxanthin effectively protects retinal photoreceptor cells from the oxidative stress caused by high glucose levels, as demonstrated in research conducted by Lai et al [95].

Lutein, a xanthophyll carotenoid found in egg yolk and leafy vegetables, possesses antioxidant properties by scavenging ROS like lipid peroxy radicals and singlet oxygen [96]. Relevant studies have demonstrated that lutein can effectively suppress oxidative stress-induced apoptosis of RGCs and RPEs, as well as protect the inner retina from ischemia-reperfusion injury due to its capacity to scavenge endogenous ROS [97].

### 4.7. Proteins

Superoxide dismutase-3 (SOD3) is a Cu/Zn-containing homotetrameric superoxide dismutase that is secreted and mostly localized on cell surfaces and in the extracellular matrix (ECM). In order to anchor itself to the ECM, it interacts with collagen and heparan sulfate proteoglycans (HSPGs) [98]. Notably, SOD enzymes regulate the levels of a variety of ROS, minimizing their potential toxicity and regulating important cellular processes that are governed by their signaling activities [99]. SOD3 functions as an antioxidant in the human vitreous, where it is concentrated in a special matrix structure that opposes the inner retina in the vitreous base and cortex. Wert et al. reported that SOD3 antioxidant dysregulation or deficiency in the vitreous base and cortex leads to elevated tissue damage and oxidative stress in the inner retina, thereby contributing to the cause of DR [100].

Transforming growth factor-beta (TGF-β) is a family of multifunctional regulatory proteins with structural similarities that exert pleiotropic effects on different organs. TGF-β is thought to possess an antioxidative cytoprotective action against cellular injury in certain kinds of cell types. Chen et al. indicated that TGF-β promotes the activation of antioxidant pathways, thereby protecting RGCs from hyperglycemia-induced harm and indicating that it could serve as an anti-diabetic treatment for DR [101].

The neuroprotective effects of glucagon-like peptide 1 (GLP-1) on the nervous system, including the retina, suggest its potential as a preventive or therapeutic agent for retinal neurodegeneration in DR. Increasing evidence further supports the advantageous impacts of GLP-1 in experimental DR [102]. Ramos et al. demonstrated that GLP-1 regulates the antioxidant defense system in the diabetic retina, exerting a neuroprotective effect that promotes neuronal proliferation and DNA repair [103].

The pleckstrin homology domain and leucine-rich repeat protein phosphatase 1 (PHLPP1) belongs to the PHLPP protein family, which exerts a pivotal influence on various physiological and pathological processes [104]. A study has suggested that PHLPP1 is essential for influencing cell viability by reducing oxidative stress and regulating the Nrf2 signaling pathways during diverse pathogenic processes [105]. Zhang et al. demonstrated that the inhibition of PHLPP1 stimulates Nrf2/ARE signaling, thereby protecting RGCs from oxidative stress and apoptosis induced by high glucose levels. This work suggests that PHLPP1 may improve high glucose-induced damage to retinal ganglion cells during the onset and progression of DR [106].

### 4.8. Other antioxidants

Capsaicin is a main active compound in chili and serves as a transient receptor potential vanillic 1 (TRPV1) agonist. It has anti-inflammatory, cardiovascular-protective and antioxidant effects and activates TRPV1 [107]. An earlier investigation showed that upregulation of CGRP by CAP can reduce apoptosis in diabetic retinal cells [108]. Our laboratory has recently reported that CAP may ameliorate DR by activating TRPV1 and suppressing the PPARγ/poldip2/Nox4/H_2_O_2_ regulatory pathway. The results of their study imply that capsaicin may serve as a novel therapeutic option for DR in clinical trials [109].

Captopril is an organic compound normally utilized for the management hypertension and as a prophylactic measure against DR. Previous research suggested that angiotensin II plays a major role in the mechanism of action of captopril in DR [110, 111]. Our laboratory has revealed through in vivo and in vitro experiments that captopril can slow the progression of DR by reducing oxidative stress [112].

The synthesized phenolic antioxidant Tert-butylhydroquinone (tBHQ) is extensively utilized as a food preservative to prolong the shelf life of food products [113]. Accumulating evidence indicated that tBHQ effectively prevents cell disturbance caused by OS inducers [114]. Notably, the recent study has demonstrated that tBHQ activates the PI3 K/Akt/eNOS pathway to prevent DR-induced OS, providing preclinical evidence for future prevention and treatment of DR. In the future, it has potential as both a treatment and prevention for DR due to its antioxidant properties [115].

Paeonol, a phenolic metabolite found in plants, has been shown to be effective in treating various diseases [116]. Liu and coworkers found that paeonol has both antidiabetic and antioxidant effects in diabetic rats induced by streptozotocin (STZ), as evidenced by a decrease in blood glucose levels with paeonol treatment [117]. Recently, Adki et al. discovered that paeonol attenuates STZ-induced DR in rats through regulating oxidative stress and the polyol pathway [118].

Geniposide (GEN) is a native extract from the fruit of gardenia with various biological properties, including anti-inflammatory and antioxidant effects [119]. Findings have shown that GEN protects diabetic rats’ myocardium from damage caused by myocardial ischemia reperfusion by preventing oxidative stress through the Nrf2/HO-1 signal pathway [120, 121]. Tu et al. subsequently demonstrated that GEN can alleviate hyperglycemia-induced oxidative stress and inflammation in DR by activating the Nrf2 signaling pathway, indicating its potential as an effective therapeutic approach for DR treatment [122].

Melatonin, also known as N-acetyl-5-methoxytryptamine, is mainly produced in the pineal gland and is essential in regulating various physiological processes [123]. The study has demonstrated a decrease in serum melatonin levels in DM patients, suggesting its potential involvement in the pathogenesis of DR [124]. Following these discoveries, Tu et al. also found that melatonin can mitigate inflammation and oxidative stress in Müller cells of DR through activation of the Sirt1 pathway [125]. Furthermore, there are many other compounds that also have some antioxidant properties. Table 1 summarizes antioxidant therapies for DR caused by oxidative stress.

**Table 1.** Application of antioxidant therapeutic strategies for DR.

Compounds	Target	Research Type	References
Gabapentin	BCATc	Animal experiment (Rat)	[39]
Fenofibrate	PPAR-α	Cell culture	[41]
Sulodexide	NOX4/NOX5	Animal experiment (Pig)	[45]
Bilobalide	Nrf2/HO-1	Animal experiment (Rat)	[49]
Carvedilol	Nrf2/ARE	Cell culture	[52]
Conbercept	NOX1/NOX4	Animal experiment (Mouse)	[55]
Scutellarin	Nrf2	Animal experiment (Mouse)	[58]
Cilostazol	Free radicals	Animal experiment (Rat)	[60]
Quercetin	Free radicals	Animal experiment (Rat)	[63]
Coumestrol	Sirt1	Animal experiment (Rat)	[66]
Dihydromyricetin	Poldip2/NOX4	Animal experiment (Rat)	[69]
Tricin	Sestrin2/Nrf2	Animal experiment (Rat)	[72]
Curcumin	Nrf2	Animal experiment (Rat)	[75]
Proanthocyanidins	Free radicals	Cell culture	[79]
Isoliquiritigenin	Sirt1	Animal experiment (Rat)	[80]
Rhaponticin	Nrf2/HO-1	Animal experiment (Rat)	[81]
Kaempferol	Bax/Bcl-2	Animal experiment (Rat)	[83]
LPC	TLR4/NF-κB	Cell culture	[87]
omega-3	Free radicals	Animal experiment (Rat)	[88]
FA	Homocysteine	Animal experiment (Mouse)	[90]
Vitamin C	Free radicals	Animal experiment (Rat)	[19]
Vitamin D	Free radicals	Cell culture	[92]
Vitamin E	Malondialdehyde	Clinical trial	[93]
Zeaxanthin	NOX4	Animal experiment (Rat)	[18]
Astaxanthin	PI3K/Akt/Nrf2	Cell culture	[95]
Lutein	Free radicals	Animal experiment (Mouse)	[97]
SOD3	Free radicals	Animal experiment (Mouse)	[100]
TGF-β	Nrf2/HO-1	Cell culture	[101]
GLP-1	MnSOD/CuZnSOD	Animal experiment (Mouse)	[103]
PHLPP1	Nrf2/ARE	Animal experiment (Rat)	[106]
Capsaicin	Poldip2	Animal experiment (Rat)	[109]
Captopril	Free radicals	Animal experiment (Rat)	[112]
tBHQ	PI3K/Akt/eNOS	Animal experiment (Rat)	[115]
Paeonol	Free radicals	Animal experiment (Rat)	[118]
Geniposide	Nrf2	Animal experiment (Mouse)	[122]
Melatonin	Sirt1	Animal experiment (Mouse)	[125]

## 5. Conclusions and future perspectives

In both type 1 and type 2 diabetes, oxidative stress plays a crucial role in the etiology of DR. It has been considered a contributor in the onset and progression of DR, and may be regarded as the primary initiator and prevalent etiological factor among the multiple molecular mechanisms involved with DR [126]. Notably, DR is associated with ROS by regulating signaling pathways involved in apoptosis and stress. DR is also a multifactorial and progressive disease characterized by the accumulation of neuronal and vascular damage, resulting in microaneurysms, neurodegeneration, edemas, neovascularization, BRB breakage and hemorrhages. It is currently one of the main global contributors to vision loss and blindness [127]. Oxidative stress is a key factor in DR development. Furthermore, an imbalance between the production and elimination of free radicals contributes to the condition known as oxidative stress [128, 129]. Thus, oxidative stress has been one of the most studied therapeutic approaches for DR [130]. Due to its high capacity for oxygen uptake, the retina is particularly vulnerable to oxidative stress in diabetes. The diabetic environment promotes oxidative stress, and molecules or medicines with antioxidant properties could be suitable candidates for attenuating DR [72].

These research’ findings can facilitate the development of cost-effective therapeutic agents for preventing and treating DR. Therefore, antioxidative intervention is imperative and pressing for DR, offering a glimmer of hope to enhance patients’ quality of life by improving functional vision. Currently, animal experiments provide most of the evidence, but their clinical therapeutic outcomes are unclear. As a result, further studies are necessary in the future.

## Author contributions

JG: Conceptualization and original draft writing; LT: Reviewing and editing of the written work; ZJ: Conceptualization, reviewing, and editing of the manuscript. All authors contributed to revising the manuscript, reading it thoroughly, and approving its final version.

## Data Availability

Data will be made available upon reasonable request from the corresponding author.

## References

[1] Standl E, Khunti K, Hansen TB, et al. The global epidemics of diabetes in the 21st century: current situation and perspectives. Eur J Prev Cardiol. 2019 Dec;26(2_suppl):7–14. doi:10.1177/2047487319881021. PubMed PMID: 31766915; eng. 31766915

[2] Basiak-Rasała A, Różańska D, Zatońska K. Food groups in dietary prevention of type 2 diabetes. Rocz Panstw Zakl Hig. 2019;70(4):347–357. doi:10.32394/rpzh.2019.0086. PubMed PMID: 31960666; eng. 31960666

[3] Cho NH, Shaw JE, Karuranga S, et al. IDF diabetes atlas: global estimates of diabetes prevalence for 2017 and projections for 2045. Diabetes Res Clin Pract. 2018 Apr;138:271–281. doi:10.1016/j.diabres.2018.02.023. PubMed PMID: 29496507; eng. 29496507

[4] Banday MZ, Sameer AS, Nissar S. Pathophysiology of diabetes: an overview. Avicenna J Med. 2020 Oct-Dec;10(4):174–188. doi:10.4103/ajm.ajm_53_20. PubMed PMID: 33437689; PubMed Central PMCID: PMCPMC7791288. eng. 33437689 PMC7791288

[5] Wang MH, Hsiao G, Al-Shabrawey M. Eicosanoids and oxidative stress in diabetic retinopathy. Antioxidants (Basel, Switzerland). 2020 Jun 12;9(6):1111. doi:10.3390/antiox9060520. PubMed PMID: 32545552; PubMed Central PMCID: PMCPMC7346161. eng. 32545552 PMC7346161

[6] Forman HJ, Zhang H. Targeting oxidative stress in disease: promise and limitations of antioxidant therapy. Nat Rev Drug Discovery. 2021; 2021/09/01, 20(9):689–709. doi:10.1038/s41573-021-00233-1 34194012 PMC8243062

[7] Guzman DC, Olguín HJ, García EH, et al. Mechanisms involved in the development of diabetic retinopathy induced by oxidative stress. Redox Report: Communications in Free Radical Research. 2017 Jan;22(1):10–16. doi:10.1080/13510002.2016.1205303. PubMed PMID: 27420399; PubMed Central PMCID: PMCPMC6837689. eng. 27420399 PMC6837689

[8] Williams M, Hogg RE, Chakravarthy U. Antioxidants and diabetic retinopathy. Curr Diab Rep. 2013 Aug;13(4):481–487. doi:10.1007/s11892-013-0384-x. PubMed PMID: 23649947; eng. 23649947

[9] Naruse R, Suetsugu M, Terasawa T, et al. Oxidative stress and antioxidative potency are closely associated with diabetic retinopathy and nephropathy in patients with type 2 diabetes. Saudi Med J. 2013 Feb;34(2):135–141. PubMed PMID: 23396458; eng. 23396458

[10] Hirsch GE, Heck TG. Inflammation: oxidative stress and altered heat shock response in type 2 diabetes: the basis for new pharmacological and non-pharmacological interventions. Arch Physiol Biochem. 2022 Apr;128(2):411–425. doi:10.1080/13813455.2019.1687522. PubMed PMID: 31746233; eng. 31746233

[11] Kumar B, Gupta SK, Srinivasan BP, et al. Hesperetin rescues retinal oxidative stress, neuroinflammation and apoptosis in diabetic rats. Microvasc Res. 2013 May;87:65–74. doi:10.1016/j.mvr.2013.01.002. PubMed PMID: 23376836; eng. 23376836

[12] Mondal LK, Bhaduri G, Bhattacharya B. Biochemical scenario behind initiation of diabetic retinopathy in type 2 diabetes mellitus. Indian J Ophthalmol. 2018 Apr;66(4):535–540. doi:10.4103/ijo.IJO_1121_17. PubMed PMID: 29582815; PubMed Central PMCID: PMCPMC5892057. eng. 29582815 PMC5892057

[13] Jian Q, Wu Y, Zhang F. Metabolomics in diabetic retinopathy: from potential biomarkers to molecular basis of oxidative stress. Cells. 2022 Sep 26;11(19). doi:10.3390/cells11193005. PubMed PMID: 36230967; PubMed Central PMCID: PMCPMC9563658. eng. PMC9563658 36230967

[14] Wu MY, Yiang GT, Lai TT, et al. The oxidative stress and mitochondrial dysfunction during the pathogenesis of diabetic retinopathy. Oxid Med Cell Longevity. 2018;2018:3420187. doi:10.1155/2018/3420187. PubMed PMID: 30254714; PubMed Central PMCID: PMCPMC6145164. eng. PMC6145164 30254714

[15] Teo ZL, Tham YC, Yu M, et al. Global prevalence of diabetic retinopathy and projection of burden through 2045: systematic review and meta-analysis. Ophthalmology. 2021 Nov;128(11):1580–1591. doi:10.1016/j.ophtha.2021.04.027. PubMed PMID: 33940045; eng. 33940045

[16] Avidor D, Loewenstein A, Waisbourd M, et al. Cost-effectiveness of diabetic retinopathy screening programs using telemedicine: a systematic review. Cost Eff Resour Alloc. 2020;18(16). doi:10.1186/s12962-020-00211-1. PubMed PMID: 32280309; PubMed Central PMCID: PMCPMC7137317. eng. PMC7137317 32280309

[17] Miller WP, Sunilkumar S, Dennis MD. The stress response protein REDD1 as a causal factor for oxidative stress in diabetic retinopathy. Free Radical Biol Med. 2021 Mar;165:127–136. doi:10.1016/j.freeradbiomed.2021.01.041. PubMed PMID: 33524531; PubMed Central PMCID: PMCPMC7956244. eng. 33524531 PMC7956244

[18] Kang Q, Yang C. Oxidative stress and diabetic retinopathy: molecular mechanisms, pathogenetic role and therapeutic implications. Redox Biol. 2020 Oct;37:101799. doi:10.1016/j.redox.2020.101799. PubMed PMID: 33248932; PubMed Central PMCID: PMCPMC7767789. eng. 33248932 PMC7767789

[19] Li C, Miao X, Li F, et al. Oxidative stress-related mechanisms and antioxidant therapy in diabetic retinopathy. Oxid Med Cell Longevity. 2017;2017:9702820. doi:10.1155/2017/9702820. PubMed PMID: 28265339; PubMed Central PMCID: PMCPMC5317113 publication of this paper. eng. PMC5317113 28265339

[20] Calderon GD, Juarez OH, Hernandez GE, et al. Oxidative stress and diabetic retinopathy: development and treatment. Eye (London, England). 2017 Aug;31(8):1122–1130. doi:10.1038/eye.2017.64. PubMed PMID: 28452994; PubMed Central PMCID: PMCPMC5558229. eng. 28452994 PMC5558229

[21] Thomas RL, Halim S, Gurudas S, et al. IDF diabetes atlas: a review of studies utilising retinal photography on the global prevalence of diabetes related retinopathy between 2015 and 2018. Diabetes Res Clin Pract. 2019 Nov;157:107840. doi:10.1016/j.diabres.2019.107840. PubMed PMID: 31733978; eng. 31733978

[22] Rivera JC, Dabouz R, Noueihed B, et al. Ischemic retinopathies: oxidative stress and inflammation. Oxid Med Cell Longevity. 2017;2017:3940241. doi:10.1155/2017/3940241. PubMed PMID: 29410732; PubMed Central PMCID: PMCPMC5749295. eng. PMC5749295 29410732

[23] Zhang J, Zhang J, Zhang C, et al. Diabetic macular edema: current understanding, molecular mechanisms and therapeutic implications. Cells. 2022 Oct 25;11(21). doi:10.3390/cells11213362. PubMed PMID: 36359761; PubMed Central PMCID: PMCPMC9655436. eng. PMC9655436 36359761

[24] Chang KC, Liu PF, Chang CH, et al. The interplay of autophagy and oxidative stress in the pathogenesis and therapy of retinal degenerative diseases. Cell Biosci. 2022 Jan 3;12(1):1. doi:10.1186/s13578-021-00736-9. PubMed PMID: 34980273; PubMed Central PMCID: PMCPMC8725349. eng. 34980273 PMC8725349

[25] Santos FM, Mesquita J, Castro-de-Sousa JP, et al. Vitreous humor proteome: targeting oxidative stress, inflammation, and neurodegeneration in vitreoretinal diseases. Antioxidants (Basel, Switzerland). 2022 Mar 6;11(3). doi:10.3390/antiox11030505. PubMed PMID: 35326156; PubMed Central PMCID: PMCPMC8944522. eng. PMC8944522 35326156

[26] Sies H. Oxidative stress: a concept in redox biology and medicine. Redox Biol. 2015;4:180–183. doi:10.1016/j.redox.2015.01.002. PubMed PMID: 25588755; PubMed Central PMCID: PMCPMC4309861. eng. 25588755 PMC4309861

[27] Nebbioso M, Franzone F, Lambiase A, et al. Oxidative stress implication in retinal diseases-a review. Antioxidants (Basel, Switzerland). 2022 Sep 10;11(9). doi:10.3390/antiox11091790. PubMed PMID: 36139862; PubMed Central PMCID: PMCPMC9495599. eng. PMC9495599 36139862

[28] Masuda T, Shimazawa M, Hara H. Retinal diseases associated with oxidative stress and the effects of a free radical scavenger (Edaravone). Oxid Med Cell Longevity. 2017;2017:9208489. doi:10.1155/2017/9208489. PubMed PMID: 28194256; PubMed Central PMCID: PMCPMC5286467. eng. PMC5286467 28194256

[29] Ola MS, Al-Dosari D, Alhomida AS. Role of oxidative stress in diabetic retinopathy and the beneficial effects of flavonoids. Curr Pharm Des. 2018;24(19):2180–2187. doi:10.2174/1381612824666180515151043. PubMed PMID: 29766782; eng. 29766782

[30] Dehdashtian E, Mehrzadi S, Yousefi B, et al. Diabetic retinopathy pathogenesis and the ameliorating effects of melatonin; involvement of autophagy, inflammation and oxidative stress. Life Sci. 2018 Jan 15;193:20–33. doi:10.1016/j.lfs.2017.12.001. PubMed PMID: 29203148; eng. 29203148

[31] Hammes HP. Diabetic retinopathy: hyperglycaemia,: oxidative stress and beyond. Diabetologia. 2018 Jan;61(1):29–38. doi:10.1007/s00125-017-4435-8. PubMed PMID: 28942458; eng. 28942458

[32] Cecilia OM, José Alberto CG, José NP, et al. Oxidative stress as the main target in diabetic retinopathy pathophysiology. J Diabetes Res. 2019;2019:8562408. doi:10.1155/2019/8562408. PubMed PMID: 31511825; PubMed Central PMCID: PMCPMC6710812. eng. 31511825 PMC6710812

[33] Satari M, Aghadavod E, Mobini M, et al. Association between miRNAs expression and signaling pathways of oxidative stress in diabetic retinopathy. J Cell Physiol. 2019 Jun;234(6):8522–8532. doi:10.1002/jcp.27801. PubMed PMID: 30478922; eng. 30478922

[34] Luo X, Wu J, Jing S, et al. Hyperglycemic stress and carbon stress in diabetic glucotoxicity. Aging Dis. 2016 Jan;7(1):90–110. doi:10.14336/ad.2015.0702. PubMed PMID: 26816666; PubMed Central PMCID: PMCPMC4723237. eng. 26816666 PMC4723237

[35] Sies H, Jones DP. Reactive oxygen species (ROS) as pleiotropic physiological signalling agents. Nat Rev Mol Cell Biol. 2020 2020/07/01;21(7):363–383. doi:10.1038/s41580-020-0230-3 32231263

[36] Ung L, Pattamatta U, Carnt N, et al. Oxidative stress and reactive oxygen species: a review of their role in ocular disease. Clin Sci (London, England: 1979). 2017 Dec 15;131(24):2865–2883. doi:10.1042/cs20171246. PubMed PMID: 29203723; eng. 29203723

[37] Koopman WJ, Nijtmans LG, Dieteren CE, et al. Mammalian mitochondrial complex I: biogenesis, regulation, and reactive oxygen species generation. Antioxid Redox Signaling. 2010 Jun 15;12(12):1431–1470. doi:10.1089/ars.2009.2743. PubMed PMID: 19803744; eng. 19803744

[38] Kim YS, Chang HK, Lee JW, et al. Protective effect of gabapentin on N-methyl-D-aspartate-induced excitotoxicity in rat hippocampal CA1 neurons. J Pharmacol Sci. 2009 Jan;109(1):144–147. doi:10.1254/jphs.08067sc. PubMed PMID: 19151547; eng. 19151547

[39] Ola MS, Alhomida AS, LaNoue KF. Gabapentin attenuates oxidative stress and apoptosis in the diabetic rat retina. Neurotox Res. 2019 Jul;36(1):81–90. doi:10.1007/s12640-019-00018-w. PubMed PMID: 30830678; eng. 30830678

[40] Keech AC, Mitchell P, Summanen PA, et al. Effect of fenofibrate on the need for laser treatment for diabetic retinopathy (FIELD study): a randomised controlled trial. Lancet (London, England). 2007 Nov 17;370(9600):1687–1697. doi:10.1016/s0140-6736(07)61607-9. PubMed PMID: 17988728; eng. 17988728

[41] Hsu YJ, Lin CW, Cho SL, et al. Protective effect of fenofibrate on oxidative stress-induced apoptosis in retinal-choroidal vascular endothelial cells: implication for diabetic retinopathy treatment. Antioxidants (Basel, Switzerland). 2020 Aug 5;9(8). doi:10.3390/antiox9080712. PubMed PMID: 32764528; PubMed Central PMCID: PMCPMC7464418. eng. PMC7464418 32764528

[42] Lauver DA, Booth EA, White AJ, et al. Sulodexide attenuates myocardial ischemia/reperfusion injury and the deposition of C-reactive protein in areas of infarction without affecting hemostasis. J Pharmacol Exp Ther. 2005 Feb;312(2):794–800. doi:10.1124/jpet.104.075283. PubMed PMID: 15365091; eng. 15365091

[43] Broekhuizen LN, Lemkes BA, Mooij HL, et al. Effect of sulodexide on endothelial glycocalyx and vascular permeability in patients with type 2 diabetes mellitus. Diabetologia. 2010 Dec;53(12):2646–2655. doi:10.1007/s00125-010-1910-x. PubMed PMID: 20865240; PubMed Central PMCID: PMCPMC2974920. eng. 20865240 PMC2974920

[44] Jo H, Jung SH, Kang J, et al. Sulodexide inhibits retinal neovascularization in a mouse model of oxygen-induced retinopathy. BMB Rep. 2014 Nov;47(11):637–642. doi:10.5483/bmbrep.2014.47.11.009. PubMed PMID: 24602608; PubMed Central PMCID: PMCPMC4281343. eng. 24602608 PMC4281343

[45] Dauth A, Bręborowicz A, Ruan Y, et al. Sulodexide prevents hyperglycemia-induced endothelial dysfunction and oxidative stress in porcine retinal arterioles. Antioxidants (Basel, Switzerland). 2023 Feb 6;12(2). doi:10.3390/antiox12020388. PubMed PMID: 36829947; PubMed Central PMCID: PMCPMC9952154. eng. PMC9952154 36829947

[46] Lu J, Xie L, Liu K, et al. Bilobalide: a review of its pharmacology, pharmacokinetics, toxicity, and safety. Phytother Res. 2021 Nov;35(11):6114–6130. doi:10.1002/ptr.7220. PubMed PMID: 34342079; eng. 34342079

[47] Yu H, Dong L-H, Zhang Y, et al. A network pharmacology-based strategy for predicting the protective mechanism of Ginkgo biloba on damaged retinal ganglion cells. Chin J Nat Med. 2022 2022/01/01/;20(1):54–66. doi:10.1016/S1875-5364(21)60109-7 35101250

[48] Cheng D, Liang B, Li Y. Antihyperglycemic effect of Ginkgo biloba extract in streptozotocin-induced diabetes in rats. BioMed Res Int. 2013;2013:162724. doi:10.1155/2013/162724. PubMed PMID: 23509685; PubMed Central PMCID: PMCPMC3591163. eng. 23509685 PMC3591163

[49] Su Q, Dong J, Zhang D, et al. Protective effects of the bilobalide on retinal oxidative stress and inflammation in streptozotocin-induced diabetic rats. Appl Biochem Biotechnol. 2022 Dec;194(12):6407–6422. doi:10.1007/s12010-022-04012-5. PubMed PMID: 35932369; eng. 35932369

[50] Arab HH, El-Sawalhi MM. Carvedilol alleviates adjuvant-induced arthritis and subcutaneous air pouch edema: modulation of oxidative stress and inflammatory mediators. Toxicol Appl Pharmacol. 2013 Apr 15;268(2):241–248. doi:10.1016/j.taap.2013.01.019. PubMed PMID: 23360886; eng. 23360886

[51] Amirshahrokhi K, Zohouri A. Carvedilol prevents pancreatic β-cell damage and the development of type 1 diabetes in mice by the inhibition of proinflammatory cytokines,: NF-κB, COX-2, iNOS and oxidative stress. Cytokine. 2021 Feb;138:155394. doi:10.1016/j.cyto.2020.155394. PubMed PMID: 33310423; eng. 33310423

[52] Zhang Y, Li M, Wang W, et al. Carvedilol activates nuclear factor E2-related factor 2/ antioxidant response element pathway to inhibit oxidative stress and apoptosis of retinal pigment epithelial cells induced by high glucose. Bioengineered. 2022 Jan;13(1):735–745. doi:10.1080/21655979.2021.2012627. PubMed PMID: 34898371; PubMed Central PMCID: PMCPMC8805944. eng. 34898371 PMC8805944

[53] Mao JB, Wu HF, Chen YQ, et al. Effect of intravitreal conbercept treatment before vitrectomy in proliferative diabetic retinopathy [Article]. Int J Ophthalmol. 2018;11(7):1217–1221. doi:10.18240/ijo.2018.07.23 30046542 PMC6048339

[54] Wang Q, Li T, Wu Z, et al. Novel VEGF decoy receptor fusion protein conbercept targeting multiple VEGF isoforms provide remarkable anti-angiogenesis effect in vivo. PLoS One. 2013;8(8):e70544. doi:10.1371/journal.pone.0070544. PubMed PMID: pub.1001768032. 23950958 PMC3741282

[55] Xia JP, Liu SQ, Wang S. Intravitreal conbercept improves outcome of proliferative diabetic retinopathy through inhibiting inflammation and oxidative stress. Life Sci. 2021 Jan 15;265:118795. doi:10.1016/j.lfs.2020.118795. PubMed PMID: 33227274; eng. 33227274

[56] Wang Z, Yu J, Wu J, et al. Scutellarin protects cardiomyocyte ischemia–reperfusion injury by reducing apoptosis and oxidative stress [Article]. Life Sci. 2016;157:200–207. doi:10.1016/j.lfs.2016.01.018 26775564

[57] Mei XY, Zhou LY, Zhang TY, et al. Scutellaria barbata attenuates diabetic retinopathy by preventing retinal inflammation and the decreased expression of tight junction protein [Article]. Int J Ophthalmol. 2017;10(6):870–877. doi:10.18240/ijo.2017.06.07 28730076 PMC5515144

[58] Mei X, Zhang T, Ouyang H, et al. Scutellarin alleviates blood-retina-barrier oxidative stress injury initiated by activated microglia cells during the development of diabetic retinopathy. Biochem Pharmacol. 2019 Jan;159:82–95. doi:10.1016/j.bcp.2018.11.011. PubMed PMID: 30447218; eng. 30447218

[59] Omi H, Okayama N, Shimizu M, et al. Cilostazol inhibits high glucose-mediated endothelial-neutrophil adhesion by decreasing adhesion molecule expression via NO production. Microvasc Res. 2004 2004/09/01/;68(2):119–125. doi:10.1016/j.mvr.2004.05.002 15313121

[60] Yeh PT, Huang YH, Chang SW, et al. Cilostazol attenuates retinal oxidative stress and inflammation in a streptozotocin-induced diabetic animal model. Curr Eye Res. 2019 Mar;44(3):294–302. doi:10.1080/02713683.2018.1542734. PubMed PMID: 30373407; eng. 30373407

[61] Hsu MY, Hsiao YP, Lin YT, et al. Quercetin alleviates the accumulation of superoxide in sodium iodate-induced retinal autophagy by regulating mitochondrial reactive oxygen species homeostasis through enhanced deacetyl-SOD2 via the Nrf2-PGC-1α-Sirt1 pathway. Antioxidants (Basel, Switzerland). 2021 Jul 14;10(7). doi:10.3390/antiox10071125. PubMed PMID: 34356358; PubMed Central PMCID: PMCPMC8301007. eng. PMC8301007 34356358

[62] Kumar B, Gupta SK, Nag TC, et al. Retinal neuroprotective effects of quercetin in streptozotocin-induced diabetic rats. Exp Eye Res. 2014 Aug;125:193–202. doi:10.1016/j.exer.2014.06.009. PubMed PMID: 24952278; eng. 24952278

[63] Gui S, Tang W, Huang Z, et al. Ultrasmall coordination polymer nanodots Fe-Quer nanozymes for preventing and delaying the development and progression of diabetic retinopathy. Adv Funct Mater; n/a(n/a):2300261. doi:10.1002/adfm.202300261

[64] Li M, Zhang D, Ge X, et al. TRAF6-p38/JNK-ATF2 axis promotes microglial inflammatory activation. Exp Cell Res. 2019 2019/03/15/;376(2):133–148. doi:10.1016/j.yexcr.2019.02.005 30763583

[65] Seida A, El-Hefnawy H, Abou-Hussein D, et al. Evaluation of medicago sativa L. sprouts as antihyperlipidemic and antihyperglycemic agent. Pak J Pharm Sci. 2015 Nov;28(6):2061–2074. PubMed PMID: 26639479; eng. 26639479

[66] Xu Y, Zhang Y, Liang H, et al. Coumestrol mitigates retinal cell inflammation, apoptosis, and oxidative stress in a rat model of diabetic retinopathy via activation of SIRT1. Aging. 2021 Feb 1;13(4):5342–5357. doi:10.18632/aging.202467. PubMed PMID: 33536350; PubMed Central PMCID: PMCPMC7950241. eng. 33536350 PMC7950241

[67] Hua YY, Zhang Y, Gong WW, et al. Dihydromyricetin improves endothelial dysfunction in diabetic mice via oxidative stress inhibition in a SIRT3-dependent manner. Int J Mol Sci. 2020 Sep 13;21(18). doi:10.3390/ijms21186699. PubMed PMID: 32933152; PubMed Central PMCID: PMCPMC7555401. eng. PMC7555401 32933152

[68] Li W, Xiao H. Dihydromyricetin alleviates high glucose-induced oxidative stress and apoptosis in human retinal pigment epithelial cells by downregulating miR-34a expression. Diabetes Metab Syndr Obes: Targets Ther. 2021;14:387–397. doi:10.2147/dmso.S290633. PubMed PMID: 33536772; PubMed Central PMCID: PMCPMC7850407. eng. PMC7850407 33536772

[69] Gui S-Y, Wang X-C, Huang Z-H, et al. Nanoscale coordination polymer Fe-DMY downregulating Poldip2-Nox4-H2O2 pathway and alleviates diabetic retinopathy. J Pharm Anal. 2023. 2023/05/12/. doi:10.1016/j.jpha.2023.05.002 PMC10759264 38174114

[70] Shalini V, Pushpan CK GS, et al. Tricin, flavonoid from njavara reduces inflammatory responses in hPBMCs by modulating the p38MAPK and PI3K/Akt pathways and prevents inflammation associated endothelial dysfunction in HUVECs. Immunobiology. 2016 Feb;221(2):137–144. doi:10.1016/j.imbio.2015.09.016. PubMed PMID: 26514297; eng. 26514297

[71] Han JM, Kwon HJ, Jung HJ. Tricin, 4’,5,7-trihydroxy-3’,5'-dimethoxyflavone,: exhibits potent antiangiogenic activity in vitro. Int J Oncol. 2016 Oct;49(4):1497–1504. doi:10.3892/ijo.2016.3645. PubMed PMID: 27498749; eng. 27498749

[72] Yang X, Li D. Tricin attenuates diabetic retinopathy by inhibiting oxidative stress and angiogenesis through regulating Sestrin2/Nrf2 signaling. Hum Exp Toxicol. 2023 Jan-Dec;42:9603271231171642. doi:10.1177/09603271231171642. PubMed PMID: 37077025; eng. 37077025

[73] Bae MK, Kim SH, Jeong JW, et al. Curcumin inhibits hypoxia-induced angiogenesis via down-regulation of HIF-1. Oncol Rep. 2006 Jun;15(6):1557–1562. PubMed PMID: 16685395; eng. 16685395

[74] Zhong Q, Mishra M, Kowluru RA. Transcription factor Nrf2-mediated antioxidant defense system in the development of diabetic retinopathy. Invest Ophthalmol Visual Sci. 2013 Jun 6;54(6):3941–3948. doi:10.1167/iovs.13-11598. PubMed PMID: 23633659; PubMed Central PMCID: PMCPMC3676188. eng. 23633659 PMC3676188

[75] Xie T, Chen X, Chen W, et al. Curcumin is a potential adjuvant to alleviates diabetic retinal injury via reducing oxidative stress and maintaining Nrf2 pathway homeostasis. Front Pharmacol. 2021;12:796565. doi:10.3389/fphar.2021.796565. PubMed PMID: 34955862; PubMed Central PMCID: PMCPMC8702852. eng. 34955862 PMC8702852

[76] Ilhan A, Yolcu U, Oztas E, et al. Therapeutic effects of proanthocyanidin and coenzyme Q10 on nitrogen mustard-induced ocular injury. Arq Bras Oftalmol. 2018 Jun;81(3):226–231. doi:10.5935/0004-2749.20180045. PubMed PMID: 29924193; eng. 29924193

[77] Li W, Jiang Y, Sun T, et al. Supplementation of procyanidins B2 attenuates photooxidation-induced apoptosis in ARPE-19 cells. Int J Food Sci Nutr. 2016 Sep;67(6):650–659. doi:10.1080/09637486.2016.1189886. PubMed PMID: 27251367; eng. 27251367

[78] Sun Y, Xiu C, Liu W, et al. Grape seed proanthocyanidin extract protects the retina against early diabetic injury by activating the Nrf2 pathway. Exp Ther Med. 2016 Apr;11(4):1253–1258. doi:10.3892/etm.2016.3033. PubMed PMID: 27073432; PubMed Central PMCID: PMCPMC4812468. eng. 27073432 PMC4812468

[79] Li H, Li R, Wang L, et al. Proanthocyanidins attenuate the high glucose-induced damage of retinal pigment epithelial cells by attenuating oxidative stress and inhibiting activation of the NLRP3 inflammasome. J Biochem Mol Toxicol. 2021 Sep;35(9):e22845. doi:10.1002/jbt.22845. PubMed PMID: 34338401; eng. 34338401

[80] Alzahrani S, Ajwah SM, Alsharif SY, et al. Isoliquiritigenin downregulates miR-195 and attenuates oxidative stress and inflammation in STZ-induced retinal injury. Naunyn-Schmiedeberg's Arch Pharmacol. 2020 Dec;393(12):2375–2385. doi:10.1007/s00210-020-01948-5. PubMed PMID: 32699958; eng. 32699958

[81] Shi Q, Cheng Y, Dong X, et al. Effects of rhaponticin on retinal oxidative stress and inflammation in diabetes through NRF2/HO-1/NF-κB signalling. J Biochem Mol Toxicol. 2020 Nov;34(11):e22568. doi:10.1002/jbt.22568. PubMed PMID: 32662907; eng. 32662907

[82] Szewczyk K, Krzaczek T, Łopatyński T, et al. Flavonoids from jovibarba globifera (Crassulaceae) rosette leaves and their antioxidant activity. Nat Prod Res. 2014;28(19):1655–1658. doi:10.1080/14786419.2014.938335. PubMed PMID: 25032483; eng. 25032483

[83] Du W, An Y, He X, et al. Protection of kaempferol on oxidative stress-induced retinal pigment epithelial cell damage. Oxid Med Cell Longevity. 2018;2018:1610751. doi:10.1155/2018/1610751. PubMed PMID: 30584457; PubMed Central PMCID: PMCPMC6280232. eng. PMC6280232 30584457

[84] Cha MH, Lee SM, Jung J. Lysophosphatidylcholine induces expression of genes involved in cholesterol biosynthesis in THP-1 derived macrophages. Steroids. 2018 Nov;139:28–34. doi:10.1016/j.steroids.2018.09.003. PubMed PMID: 30217786; eng. 30217786

[85] Park S, Kim JA, Choi S, et al. Superoxide is a potential culprit of caspase-3 dependent endothelial cell death induced by lysophosphatidylcholine. J Physiol Pharmacol. 2010 Aug;61(4):375–381. PubMed PMID: 20814064; eng. 20814064

[86] Cheng L, Han X, Shi Y. A regulatory role of LPCAT1 in the synthesis of inflammatory lipids, PAF and LPC, in the retina of diabetic mice. Am J Physiol Endocrinol Metab. 2009 Dec;297(6):E1276-82. doi:10.1152/ajpendo.00475.2009. PubMed PMID: 19773578; PubMed Central PMCID: PMCPMC2793047. eng. 19773578 PMC2793047

[87] Zhao H, He Y. Lysophosphatidylcholine offsets the protective effects of bone marrow mesenchymal stem cells on inflammatory response and oxidative stress injury of retinal endothelial cells via TLR4/NF-κB signaling. J Immunol Res. 2021;2021:2389029. doi:10.1155/2021/2389029. PubMed PMID: 34692851; PubMed Central PMCID: PMCPMC8531799. eng. 34692851 PMC8531799

[88] Suzumura A, Terao R, Kaneko H. Protective effects and molecular signaling of n-3 fatty acids on oxidative stress and inflammation in retinal diseases. Antioxidants (Basel, Switzerland). 2020 Sep 26;9(10). doi:10.3390/antiox9100920. PubMed PMID: 32993153; PubMed Central PMCID: PMCPMC7600094. eng. PMC7600094 32993153

[89] Mao X, Xing X, Xu R, et al. Folic acid and vitamins D and B12 correlate with homocysteine in Chinese patients with type-2 diabetes mellitus, hypertension, or cardiovascular disease. Medicine (Baltimore). 2016 Feb;95(6):e2652. doi:10.1097/md.0000000000002652. PubMed PMID: 26871790; PubMed Central PMCID: PMCPMC4753885. eng. 26871790 PMC4753885

[90] Lei XW, Li Q, Zhang JZ, et al. The protective roles of folic acid in preventing diabetic retinopathy are potentially associated with suppressions on angiogenesis, inflammation, and oxidative stress. Ophthalmic Res. 2019;62(2):80–92. doi:10.1159/000499020. PubMed PMID: 31018207; eng. 31018207

[91] Valle MS, Russo C, Malaguarnera L. Protective role of vitamin D against oxidative stress in diabetic retinopathy. Diabetes Metab Res Rev. 2021 Nov;37(8):e3447. doi:10.1002/dmrr.3447. PubMed PMID: 33760363; eng. 33760363

[92] Fernandez-Robredo P, González-Zamora J, Recalde S, et al. Vitamin D protects against oxidative stress and inflammation in human retinal cells. Antioxidants (Basel, Switzerland). 2020 Sep 8;9(9). doi:10.3390/antiox9090838. PubMed PMID: 32911690; PubMed Central PMCID: PMCPMC7555517. eng. PMC7555517 32911690

[93] Chatziralli IP, Theodossiadis G, Dimitriadis P, et al. The effect of vitamin E on oxidative stress indicated by serum malondialdehyde in insulin-dependent type 2 diabetes mellitus patients with retinopathy. Open Ophthalmol J. 2017;11:51–58. doi:10.2174/1874364101711010051. PubMed PMID: 28567166; PubMed Central PMCID: PMCPMC5420190. eng. 28567166 PMC5420190

[94] Landon R, Gueguen V, Petite H, et al. Impact of astaxanthin on diabetes pathogenesis and chronic complications. Mar Drugs. 2020 Jul 9;18(7). doi:10.3390/md18070357. PubMed PMID: 32660119; PubMed Central PMCID: PMCPMC7401277. eng. PMC7401277 32660119

[95] Lai TT, Yang CM, Yang CH. Astaxanthin protects retinal photoreceptor cells against high glucose-induced oxidative stress by induction of antioxidant enzymes via the PI3K/Akt/Nrf2 pathway. Antioxidants (Basel, Switzerland). 2020 Aug 10;9(8). doi:10.3390/antiox9080729. PubMed PMID: 32785112; PubMed Central PMCID: PMCPMC7465141. eng. PMC7465141 32785112

[96] Böhm F, Edge R, Truscott TG. Interactions of dietary carotenoids with singlet oxygen (1O2) and free radicals: potential effects for human health. Acta Biochim Pol. 2012;59(1):27–30. PubMed PMID: 22428151; eng. doi:10.18388/abp.2012_2164 22428151

[97] Ahn YJ, Kim H. Lutein as a modulator of oxidative stress-mediated inflammatory diseases. Antioxidants (Basel, Switzerland). 2021 Sep 13;10(9). doi:10.3390/antiox10091448. PubMed PMID: 34573081; PubMed Central PMCID: PMCPMC8470349. eng. PMC8470349 34573081

[98] Kobylecki CJ, Afzal S, Nordestgaard BG. Genetically low antioxidant protection and risk of cardiovascular disease and heart failure in diabetic subjects. EBioMedicine. 2015 Dec;2(12):2010–2015. doi:10.1016/j.ebiom.2015.11.026. PubMed PMID: 26844281; PubMed Central PMCID: PMCPMC4703764. eng. 26844281 PMC4703764

[99] Wang Y, Branicky R, Noë A, et al. Superoxide dismutases: dual roles in controlling ROS damage and regulating ROS signaling. J Cell Biol. 2018 Jun 4;217(6):1915–1928. doi:10.1083/jcb.201708007. PubMed PMID: 29669742; PubMed Central PMCID: PMCPMC5987716. eng. 29669742 PMC5987716

[100] Wert KJ, Velez G, Cross MR, et al. Extracellular superoxide dismutase (SOD3) regulates oxidative stress at the vitreoretinal interface. Free Radical Biol Med. 2018 Aug 20;124:408–419. doi:10.1016/j.freeradbiomed.2018.06.024. PubMed PMID: 29940351; PubMed Central PMCID: PMCPMC6233711. eng. 29940351 PMC6233711

[101] Chen HY, Ho YJ, Chou HC, et al. The role of transforming growth factor-beta in retinal ganglion cells with hyperglycemia and oxidative stress. Int J Mol Sci. 2020 Sep 4;21(18). doi:10.3390/ijms21186482. PubMed PMID: 32899874; PubMed Central PMCID: PMCPMC7554964. eng. PMC7554964 32899874

[102] Hernández C, Bogdanov P, Corraliza L, et al. Topical administration of GLP-1 receptor agonists prevents retinal neurodegeneration in experimental diabetes. Diabetes. 2016 Jan;65(1):172–187. doi:10.2337/db15-0443. PubMed PMID: 26384381; eng. 26384381

[103] Ramos H, Bogdanov P, Sampedro J, et al. Beneficial effects of glucagon-like peptide-1 (GLP-1) in diabetes-induced retinal abnormalities: involvement of oxidative stress. Antioxidants (Basel, Switzerland). 2020 Sep 10;9(9). doi:10.3390/antiox9090846. PubMed PMID: 32927585; PubMed Central PMCID: PMCPMC7554849. eng. PMC7554849 32927585

[104] Hribal ML, Mancuso E, Spiga R, et al. PHLPP phosphatases as a therapeutic target in insulin resistance-related diseases. Expert Opin Ther Targets. 2016 2016/06/02;20(6):663–675. doi:10.1517/14728222.2016.1130822 26652182

[105] Qiu Y, Wu Y, Meng M, et al. GYY4137 protects against myocardial ischemia/reperfusion injury via activation of the PHLPP-1/Akt/Nrf2 signaling pathway in diabetic mice. J Surg Res. 2018 2018/05/01/;225:29–39. doi:10.1016/j.jss.2017.12.030 29605032

[106] Zhang X, Lu Y, He N, et al. Downregulation of PHLPP1 ameliorates high glucose-evoked injury in retinal ganglion cells by attenuating apoptosis and oxidative stress through enhancement of Nrf2 activation. Exp Cell Res. 2020 Dec 15;397(2):112344. doi:10.1016/j.yexcr.2020.112344. PubMed PMID: 33164862; eng. 33164862

[107] Panchal SK, Bliss E, Brown L. Capsaicin in metabolic syndrome. Nutrients. 2018 May 17;10(5). doi:10.3390/nu10050630. PubMed PMID: 29772784; PubMed Central PMCID: PMCPMC5986509. eng. PMC5986509 29772784

[108] Yang J-H, Guo Z, Zhang T, et al. STZ treatment induced apoptosis of retinal cells and effect of up-regulation of calcitonin gene related peptide in rats. J Diabetes Complicat. 2013 2013/11/01/;27(6):531–537. doi:10.1016/j.jdiacomp.2013.08.001 24051030

[109] Liu K, Gao X, Hu C, et al. Capsaicin ameliorates diabetic retinopathy by inhibiting poldip2-induced oxidative stress. Redox Biol. 2022 Oct;56:102460. doi:10.1016/j.redox.2022.102460. PubMed PMID: 36088760; PubMed Central PMCID: PMCPMC9468458. eng. 36088760 PMC9468458

[110] Senanayake PD, Bonilha VL JWP, et al. Retinal angiotensin II and angiotensin-(1-7) response to hyperglycemia and an intervention with captopril. J Renin Angiotensin Aldosterone Syst. 2018 Jul-Sep;19(3):1470320318789323. doi:10.1177/1470320318789323. PubMed PMID: 30126320; PubMed Central PMCID: PMCPMC6104213. eng. 30126320 PMC6104213

[111] Zhang JZ, Xi X, Gao L, et al. Captopril inhibits capillary degeneration in the early stages of diabetic retinopathy. Curr Eye Res. 2007 Oct;32(10):883–889. doi:10.1080/02713680701584123. PubMed PMID: 17963108; eng. 17963108

[112] Gao X, Liu K, Hu C, et al. Captopril alleviates oxidative damage in diabetic retinopathy. Life Sci. 2022 Feb 1;290:120246. doi:10.1016/j.lfs.2021.120246. PubMed PMID: 34953892; eng. 34953892

[113] Heras-Sandoval D, Pérez-Rojas JM, Hernández-Damián J, et al. The role of PI3K/AKT/mTOR pathway in the modulation of autophagy and the clearance of protein aggregates in neurodegeneration. Cell Signal. 2014 Dec;26(12):2694–2701. doi:10.1016/j.cellsig.2014.08.019. PubMed PMID: 25173700; eng. 25173700

[114] Graupera M, Potente M. Regulation of angiogenesis by PI3K signaling networks. Exp Cell Res. 2013 2013/05/15/;319(9):1348–1355. doi:10.1016/j.yexcr.2013.02.021 23500680

[115] Cao Y, Wang J, Wei F, et al. Tert-butylhydroquinone protects the retina from oxidative stress in STZ-induced diabetic rats via the PI3K/Akt/eNOS pathway. Eur J Pharmacol. 2022 Nov 15;935:175297. doi:10.1016/j.ejphar.2022.175297. PubMed PMID: 36174669; eng. 36174669

[116] Zhang L, Li DC, Liu LF. Paeonol: pharmacological effects and mechanisms of action. Int Immunopharmacol. 2019 Jul;72:413–421. doi:10.1016/j.intimp.2019.04.033. PubMed PMID: 31030097; eng. 31030097

[117] Liu J, Wang S, Feng L, et al. Hypoglycemic and antioxidant activities of paeonol and its beneficial effect on diabetic encephalopathy in streptozotocin-induced diabetic rats. J Med Food. 2013 Jul;16(7):577–586. doi:10.1089/jmf.2012.2654. PubMed PMID: 23875897; eng. 23875897

[118] Adki KM, Kulkarni YA. Paeonol attenuates retinopathy in streptozotocin-induced diabetes in rats by regulating the oxidative stress and polyol pathway. Front Pharmacol. 2022;13:891485. doi:10.3389/fphar.2022.891485. PubMed PMID: 36160440; PubMed Central PMCID: PMCPMC9490113. eng. 36160440 PMC9490113

[119] Koo HJ, Lim KH, Jung HJ, et al. Anti-inflammatory evaluation of gardenia extract, geniposide and genipin. J Ethnopharmacol. 2006 Feb 20;103(3):496–500. doi:10.1016/j.jep.2005.08.011. PubMed PMID: 16169698; eng. 16169698

[120] Hu X, Zhang X, Jin G, et al. Geniposide reduces development of streptozotocin-induced diabetic nephropathy via regulating nuclear factor-kappa B signaling pathways. Fundam Clin Pharmacol. 2017 Feb;31(1):54–63. doi:10.1111/fcp.12231. PubMed PMID: 27521287; eng. 27521287

[121] Wang GF, Wu SY, Xu W, et al. Geniposide inhibits high glucose-induced cell adhesion through the NF-kappaB signaling pathway in human umbilical vein endothelial cells. Acta Pharmacol Sin. 2010 Aug;31(8):953–962. doi:10.1038/aps.2010.83. PubMed PMID: 20686520; PubMed Central PMCID: PMCPMC4007819. eng. 20686520 PMC4007819

[122] Tu Y, Li L, Zhu L, et al. Geniposide attenuates hyperglycemia-induced oxidative stress and inflammation by activating the Nrf2 signaling pathway in experimental diabetic retinopathy. Oxid Med Cell Longevity. 2021;2021:9247947. doi:10.1155/2021/9247947. PubMed PMID: 34938383; PubMed Central PMCID: PMCPMC8687848. eng. PMC8687848 34938383

[123] Cipolla-Neto J, Amaral F. Melatonin as a hormone: new physiological and clinical insights. Endocr Rev. 2018;39(6):990–1028. doi:10.1210/er.2018-00084%J. Endocrine Reviews. 30215696

[124] Hikichi T, Tateda N, Miura T. Alteration of melatonin secretion in patients with type 2 diabetes and proliferative diabetic retinopathy [Article]. Clinical Ophthalmology. 2011;5(1):655–660. doi:10.2147/OPTH.S19559 21629571 PMC3104794

[125] Tu Y, Song E, Wang Z, et al. Melatonin attenuates oxidative stress and inflammation of müller cells in diabetic retinopathy via activating the Sirt1 pathway. Biomed Pharmacother. 2021 May;137:111274. doi:10.1016/j.biopha.2021.111274. PubMed PMID: 33517190; eng. 33517190

[126] Wu Y, Tang L, Chen B. Oxidative stress: implications for the development of diabetic retinopathy and antioxidant therapeutic perspectives. Oxid Med Cell Longevity. 2014;2014:752387. doi:10.1155/2014/752387. PubMed PMID: 25180070; PubMed Central PMCID: PMCPMC4142742. eng. PMC4142742 25180070

[127] Carpi-Santos R, de Melo Reis RA, Gomes FCA, et al. Contribution of müller cells in the diabetic retinopathy development: focus on oxidative stress and inflammation. Antioxidants (Basel, Switzerland). 2022 Mar 23;11(4). doi:10.3390/antiox11040617. PubMed PMID: 35453302; PubMed Central PMCID: PMCPMC9027671. eng. PMC9027671 35453302

[128] Poprac P, Jomova K, Simunkova M, et al. Targeting free radicals in oxidative stress-related human diseases. Trends Pharmacol Sci. 2017 2017/07/01/;38(7):592–607. doi:10.1016/j.tips.2017.04.005 28551354

[129] Prasad S, Gupta SC, Tyagi AK. Reactive oxygen species (ROS) and cancer: role of antioxidative nutraceuticals. Cancer Lett. 2017 2017/02/28/;387:95–105. doi:10.1016/j.canlet.2016.03.042 27037062

[130] Robles-Rivera RR, Castellanos-González JA, Olvera-Montaño C, et al. Adjuvant therapies in diabetic retinopathy as an early approach to delay Its progression: the importance of oxidative stress and inflammation. Oxid Med Cell Longevity. 2020;2020:3096470. doi:10.1155/2020/3096470. PubMed PMID: 32256949; PubMed Central PMCID: PMCPMC7086452. eng. PMC7086452 32256949

